# Construction and validation of a prognostic signature based on anoikis-related lncRNAs in lung adenocarcinoma

**DOI:** 10.18632/aging.205905

**Published:** 2024-06-07

**Authors:** Xiaoqi Dong, Chuan Shao, Shuguang Xu, Jinjing Tu, Wenjing Xu, Dahua Chen, Yaodong Tang

**Affiliations:** 1 Department of Pulmonary and Critical Care Medicine, Ningbo Medical Center Lihuili Hospital (Lihuili Hospital Affiliated to Ningbo University), Ningbo, China; 2 Ningbo University Health Science Center, Ningbo, China; 3 Department of Gastroenterology, Ningbo Medical Center Lihuili Hospital (Lihuili Hospital Affiliated to Ningbo University), Ningbo, China

**Keywords:** lung adenocarcinoma, lncRNAs, anoikis, signature, prognosis

## Abstract

Lung adenocarcinoma (LUAD) is the most common type of lung cancer and is characterized by a high death rate and a poor prospect for survival. Anoikis, which is a kind of programmed cell apoptosis, is an important factor in the advancement of tumors. Nonetheless, the function of anoikis-related lncRNAs (ARLRs) in LUAD is still not well understood. The TCGA database was queried for genomic and clinical information. A prognostic signature for ARLRs was established via the use of coexpression analysis and Cox regression. Validation of the model’s accuracy was conducted utilizing K-M curves and receiver operating characteristic (ROC) curves, and the signature was utilized to develop a nomogram. LncRNAs were implicated in the progression of tumors, as determined by functional enrichment analysis. There was an improvement in prognosis, increased immune cell infiltration, and higher immune scores among the low-risk patients. Additionally, we found that the two groups had varied anticancer drug sensitivities, which could help guide treatment. The impact of one ARLR, AC026355.2, on migration and invasion was validated by *in vitro* experiments in LUAD cells. Herein, a new lncRNA signature associated with anoikis was identified and estimated, potentially serving as a prognostic indicator for LUAD patients.

## INTRODUCTION

Undoubtedly, lung cancer is a prevalent malignant tumor that poses a significant risk to the well-being and life of humans, resulting in significant morbidity and mortality [1]. Non-small cell lung cancers (NSCLCs) comprise around 80% of lung cancers, with lung adenocarcinomas (LUADs) representing more than 70% of these cases [2, 3]. Nearly 40% of individuals with non-small cell lung cancer (NSCLC) exhibit distant metastases after they are first diagnosed and this number rises to 40% while undergoing therapy [4]. Nevertheless, therapies for distant metastases have limited effectiveness, leading to unsatisfactory outcomes. Even small primary tumors are susceptible to metastasis in LUAD [5]. Consequently, the 5-year survival rate for patients afflicted with advanced LUAD is below 20%, indicating an unfavorable prognosis [6]. The discovery of prognostic markers for the prediction of LUAD patients’ prognoses and survival rates is a pressing concern.

Anoikis, which is generated through cell detachment from the extracellular matrix (ECM), is an exceptional form of programmed cell apoptosis that is critically involved in the development, homeostasis and metastasis of tumors. Cancer cells, as opposed to healthy epithelial cells, are resistant to anoikis and are not dependent on ECM adhesion for survival and proliferation. The term for this acquired capability is “anoikis resistance.” Metastasis of cancer may arise from the dissemination of anoikis-resistant cancerous cells via the circulatory system to distant tissues or organs [7]. Understanding how anoikis occurs in cancer at the molecular level might have significant practical implications.

Long noncoding RNAs (lncRNAs) are a subclass of noncoding RNA distinguished by their incapacity to encode proteins and transcriptional length exceeding 200 nucleotides; lncRNAs are involved in numerous vital biological processes, including the regulation of cell differentiation, cell cycle, and epigenetic processes [8]. Various types of malignancies may exhibit abnormal lncRNA expression, and dysregulated lncRNAs may function as tumor promoters or inhibitors [9]. Extensive research has shown that tumorigenesis is intricately linked to aberrant lncRNA expression in NSCLC. It is suggested that linc00312—whose expression is reduced in NSCLC tissues—activates the HOXA5 transcription factor (TF), which is essential for cellular proliferation and tissue growth [10].

NSCLC tissues have high levels of LncRNA-UCA1, and tumor cell proliferation is inhibited by UCA1 silencing [11]. Additionally, the associated lncRNAs are considered to have a significant role in determining the prognosis of patients. In one study, NSCLC tissues were shown to have substantially lower levels of SPRY4-IT1 expression, which were significantly linked to pathological lymph node metastasis (pN) (*P* = 0.003), pathological stage (*P* < 0.001), and tumor size (*P* = 0.001); Hence, SPRY4-IT1 was shown to independently function as a robust risk predictor of NSCLC prognosis in the study [12]. Nonetheless, the involvement of lncRNAs in LUCD requires additional research.

At present, limited research has been conducted in LUAD on prognostic markers derived from anoikis-associated lncRNAs. Herein, we identified anoikis-related lncRNAs (ARLRs) using bioinformatics analysis to explore their biological functions and predict LUAD patients’ prognoses.

## MATERIALS AND METHODS

### Acquisition and processing of data

Clinical data and RNA sequencing (RNA-seq) results from 539 patients with LUAD and 59 normal controls were retrieved from The Cancer Genome Atlas (TCGA) database (last assessed on September 2022) in the FPKM format (https://portal.gdc.cancer.gov/). Overall, 37 patients were excluded from the analysis due to the absence of survival time or survival status data. By setting a relevance score of >2, 65 anoikis-related genes (ARGs) were retrieved from GeneCards [13]. We employed limma in R (|R| > 0.4 and *p*-value < 0.001) to perform Pearson correlation analysis on the coexpression of lncRNA and ARGs to discover potential ARLRs. Since our research relied on publicly available data, there were no ethical concerns.

### Generation and assessment of the ARLR signature

Two distinct groups were formed from the LUAD patients at random, with a ratio of 1:1. Table 1 displays the patients’ clinical characteristics in both the training and the testing groups. To begin, we screened the training cohort for ARLRs using differential expression and Pearson correlation analyses. Next, genes were selected using univariate Cox and Lasso-Cox regression analyses, based on the ARLRs, to reduce the probability of overfitting. Thereafter, a prognostic signature was developed utilizing multivariate Cox regression analysis, which incorporated ARLRs with *p* < 0.05. The following calculation determined the anoikis-related lncRNA signature (ARLSig) risk score: risk factor = (ARLRs 1 expression × coefficient) + (ARLRs 2 expression × coefficient) … + (ARLRs n expression × coefficient).

**Table 1:** The clinical feature of the patients with LUAD in the training and testing group.

**Type**	**Testing group *n* (%)**	**Training group *n* (%)**	***P***
Gender			
Female	134 (53.39%)	138 (54.98%)	0.7881
Male	117 (46.61%)	113 (45.02%)	
Age			
≤65	113 (45.02%)	125 (49.8%)	0.465
>65	130 (51.79%)	124 (49.4%)	
Unknow	8 (3.19%)	2 (0.8%)	
T			
T1	88 (35.06%)	80 (31.87%)	0.8169
T2	131 (52.19%)	137 (54.58%)	
T3	21 (8.37%)	24 (9.56%)	
T4	10 (3.98%)	8 (3.19%)	
Unknow	1 (0.4%)	2 (0.8%)	
*N*			
N0	170 (67.73%)	170 (67.73%)	0.5844
N1	46 (18.33%)	46 (18.33%)	
N2	30 (11.95%)	30 (11.95%)	
N3	1 (0.4%)	1 (0.4%)	
Unknow	4 (1.59%)	4 (1.59%)	
M			
M0	240 (95.62%)	234 (93.23%)	0.2955
M1	9 (3.59%)	15 (5.98%)	
Unknow	2 (0.8%)	2 (0.8%)	
Stage			
Stage I	138 (54.98%)	138 (54.98%)	0.4373
Stage II	62 (24.7%)	62 (24.7%)	
Stage III	37 (14.74%)	37 (14.74%)	
Stage IV	9 (3.59%)	9 (3.59%)	
Unknow	5 (1.99%)	5 (1.99%)	
Survival status			
Alive	164 (65.34%)	156 (62.15%)	0.5158
Death	87 (34.66%)	95 (37.85%)	

We classified the LUAD patients in the training, test, and entire cohorts as high- or low-risk as determined by the median risk score value. The pheatmap package in R was utilized to generate heatmaps, risk curves, and survival statuses for various patient sets depending on the risk score. We utilized the timeROC package to conduct a time-dependent receiver operating characteristic (ROC) curve analysis to ascertain the ARLSig’s accuracy and sensitivity. The survival package was employed to execute K-M analysis to determine overall survival (OS), progression-free survival (PFS), and disease-specific survival (DSS) in patients with varying risk scores. Univariate and multivariate analyses were conducted to establish the signature’s independent prognostic significance. Clinicopathological characteristics were also evaluated for their predictive significance in each group via K-M analysis. To determine how the patients with varied scores were distributed, principal component analysis (PCA) was carried out.

The independent parameters were subsequently utilized to develop a nomogram. To gauge the ARLSig score’s reliability, we compared the expected and actual values in the TCGA-LUAD cohort utilizing calibration and time-dependent ROC curves.

### Biological function of the risk scores

The high- and low-risk groups were compared via differential gene expression (DEGs) analysis with criteria of |log2FC| > 1 and *p* < 0.05, and volcano plots were applied to display the results. The clusterProfiler package was adopted for gene ontology (GO) functional analysis. The GSEA software v4.2.1 was also utilized for gene set enrichment analysis (GSEA), and functional enrichment was considered significant when the FDR < 0.05.

### Analysis of the risk score’s relationship with TMB

To better understand how tumor mutation burden (TMB) correlated with risk score, we developed a waterfall plot and subsequently assessed the varying TMB in the groups at high and low risk. Subsequently, the survival package was employed to generate survival curves.

### Infiltration of immune cells and anticancer treatment

A heatmap was used to display the results of the immune-related activities identified by the limma program in LUAD patients. The statuses of immune infiltration were examined utilizing the following tools: XCELL, CIBERSORT, MCP-counter, QUANTISEQ, EPIC, TIMER, and CIBERSORT-ABS. The results are displayed as a bubble diagram and are based on the infiltration estimate profile in the TCGA database. Additionally, the estimate package was utilized to ascertain the link between the TME score and the risk score.

An assessment of the response to immunotherapy was conducted by retrieving each LUAD patient’s immunophenoscore (IPS) from the TCIA database (https://tcia.at/home), which was determined by the expression of genes associated with immunity and denoting four distinct categories of immune (selected immunomodulators, effector cells, MHC molecules, and immunosuppressive cells). We employed the pRRophetic package to determine the half-maximal inhibitory concentration (IC50) to evaluate anticancer drugs for LUAD.

### Cell culture and quantitative real-time PCR (qPCR)

The human LUAD cell line and one human embryonic lung cell line, MRC-5, were both stored in our laboratory. The experimental parameters for cell culture included the following: complete RPMI-1640 medium that contained 10% fetal bovine serum (FBS) and 1% antibiotics (100 ng/mL streptomycin and 100 U/mL penicillin) in a humidified incubator at 37°C with 5% CO2. The cells were treated with TRI reagent to isolate total RNA. PrimeScript RT was employed to reverse transcribe the total RNA to acquire cDNA, followed by qPCR (Takara Bio Company). The primers used for qPCR included the following: AC026355.2, forward primer: 5′-CTGGATGCTTCCTGCCCTTGAAC-3′, reverse primer: 5′-CCAACAGCCCCTGCCAAACC-3′.

### Cell transfection

Lipofectamine 2000 was used for cell transfection in compliance with established protocols. After 48 to 72 hours, the transfected cells were harvested to use for additional experiments. In this study, a negative control siRNA (si-NC) and si-AC026355.2 were chemically synthesized by GenePharma (Shanghai, China). The sequence of si-AC026355.2 was 5′-GUGACAGGCAACACCUAUATT-3′.

### Transwell experiment

The transfected A549 cells (6 × 10^5^ cells/mL) were placed in the upper chamber in a serum-free culture medium. Thereafter, the bottom chamber of the transwell plate was gradually filled with 500 μL of a culture medium that contained 10% FBS. Following 24 hours, the infiltrating cells were fixed for a half-hour at room temperature utilizing 4% polyoxymethylene, after which 0.5% crystal violet was introduced to stain them for 10 minutes. The cell count was performed in four fields of view that were selected at random utilizing a microscope.

### Wound healing assay

Approximately 4 × 10^5^ cells per well were inoculated in 6-well plates. The next day, a sterile pipette tip was employed to create a scratch at the bottom of each well. At both the 0 and 48-hour post-scratch intervals, the area was microscopically imaged. The following equation was employed to compute the scratch healing rate: (initial scratch width - observed scratch width at the designated time point)/initial scratch width × 100%.

### Statistical analysis

R software was employed for all statistical analyses undertaken in this study. We evaluated the model’s performance via ROC analyses. The survival rates of the high- and low-risk groups were compared via K-M analysis. The tests were all two-tailed, and *p* < 0.05 was established as the significance criterion.

### Data availability statement

The data used for our analysis in this study are openly available in a public database (https://portal.gdc.cancer.gov/).

## RESULTS

### ARLRs identification of and creation of the prognostic signature

**Figure 1:**
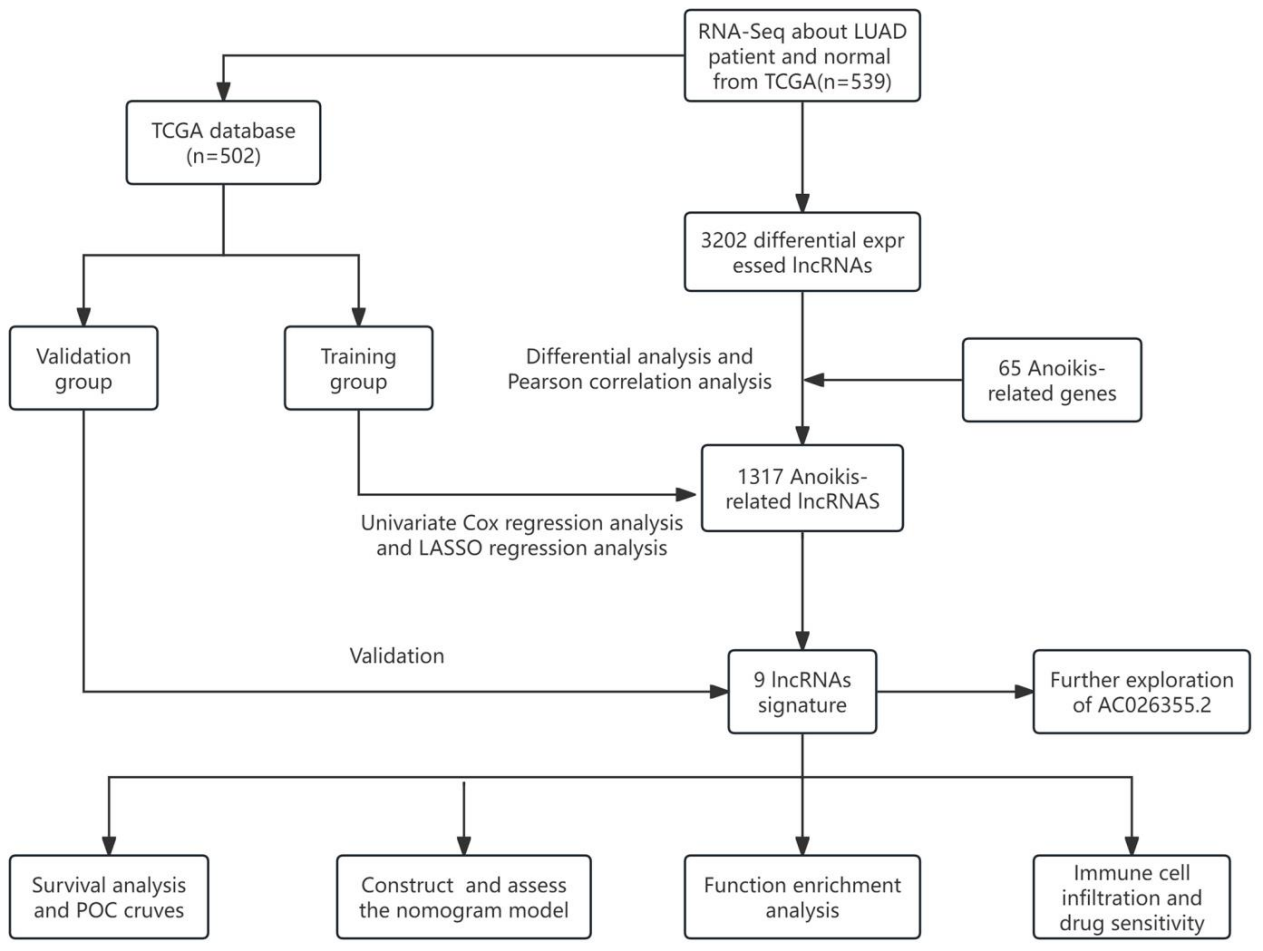
The flow overview of the study.

**Figure 2:**
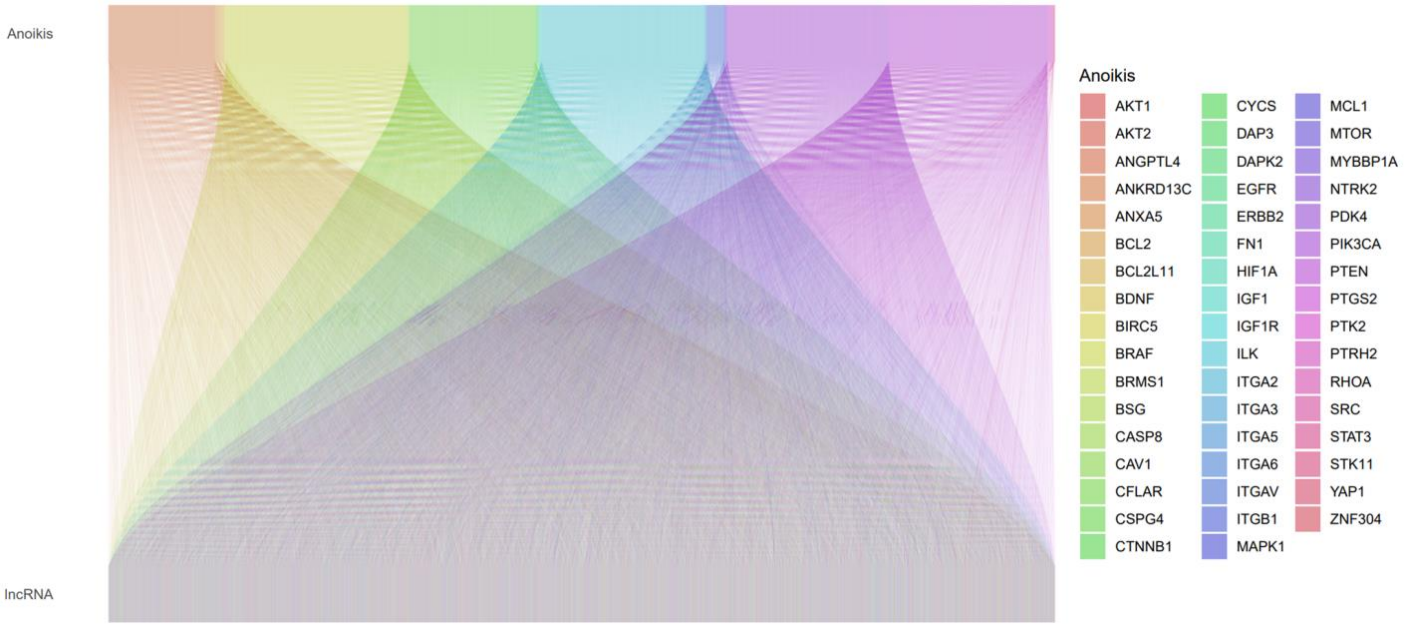
A Sankey plot illustrating the relationship between lncRNAs and anoikis-related genes (ARGs).

**Figure 3:**
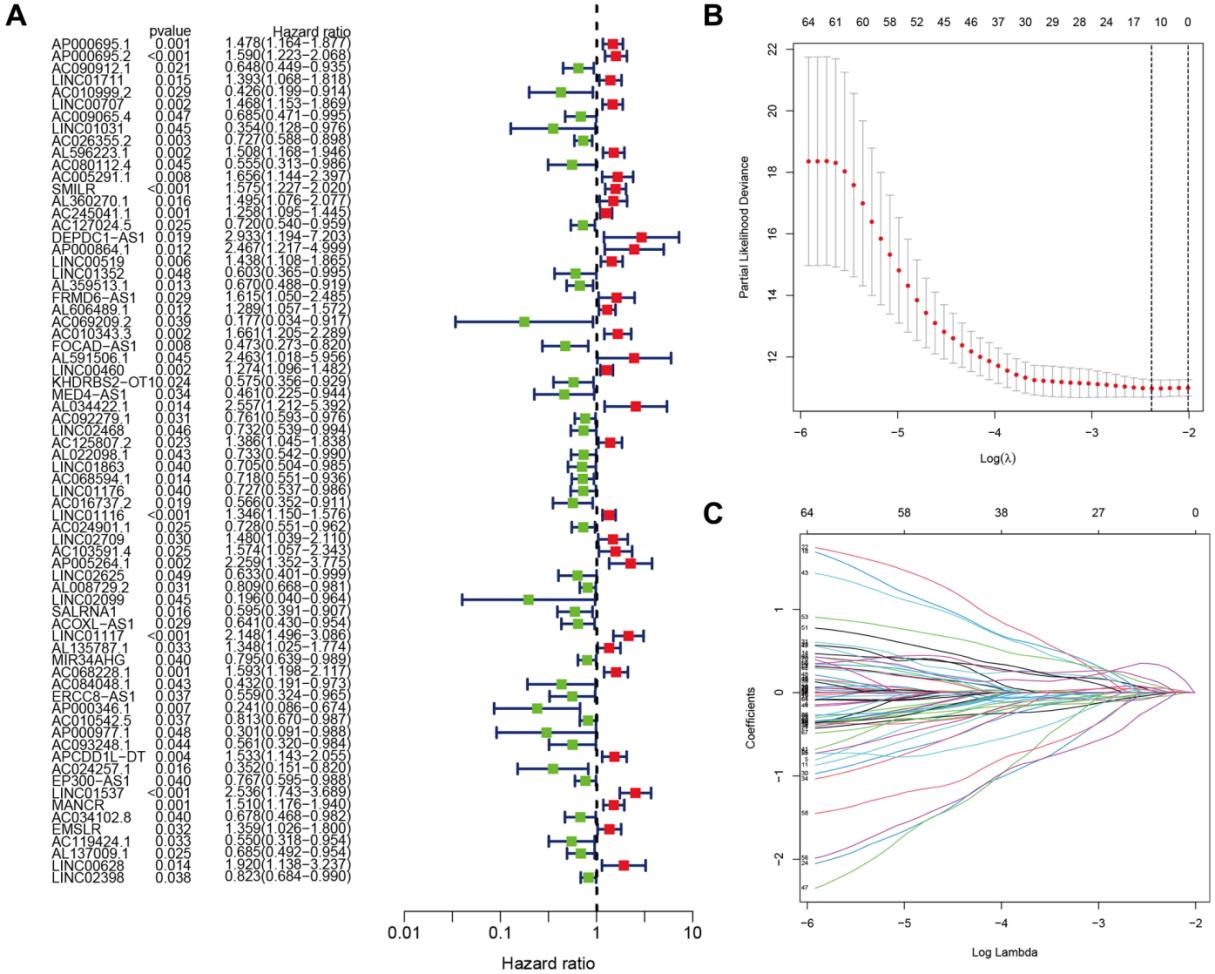
**Identification of the anoikis-related lncRNA (ARLR) signature for LUAD patients.** Various lncRNAs linked to high or low risk are shown in a forest plot according to the results of univariate Cox regression. (**A**) ARLR screening using LASSO regression at the minimal cross-validation point. (**B**) Each independent variable’s trajectory (**C**).

Figure 1 depicts a comprehensive flow overview of our study. We enrolled 59 healthy controls and 502 LUAD patients who had accessible RNA seq data, and an aggregate of 3,202 differentially expressed lncRNAs was discovered. As illustrated in Figure 2, coexpression relationships between 65 ARGs from GeneCards and differentially expressed lncRNAs are shown in the Sankey diagram. The next step was to randomly assign database patients to either a training or testing group. Table 1 displays the essential clinical characteristics of the LUAD patients at baseline. As determined by univariate and LASSO regression analyses, the prognostic signature was composed of 9 ARLRs (Figure 3). The ARLSig formula was expressed as indicated: risk score = (−0.345342588082988 × AC090912.1) + (0.251214414934825 × LINC00707) + (−0.18011074824835 × AC026355.2) + (−0.493695647381226 × FOCAD-AS1) + (0.172008983013553 × LINC00460) + (0.366834641287433 × LINC01117) + (0.4332718020797 × AC068228.1) + (−1.04234276424881 × AP000346.1) + (0.428355116775672 × LINC01537).

### ARLSig validation

**Figure 4:**
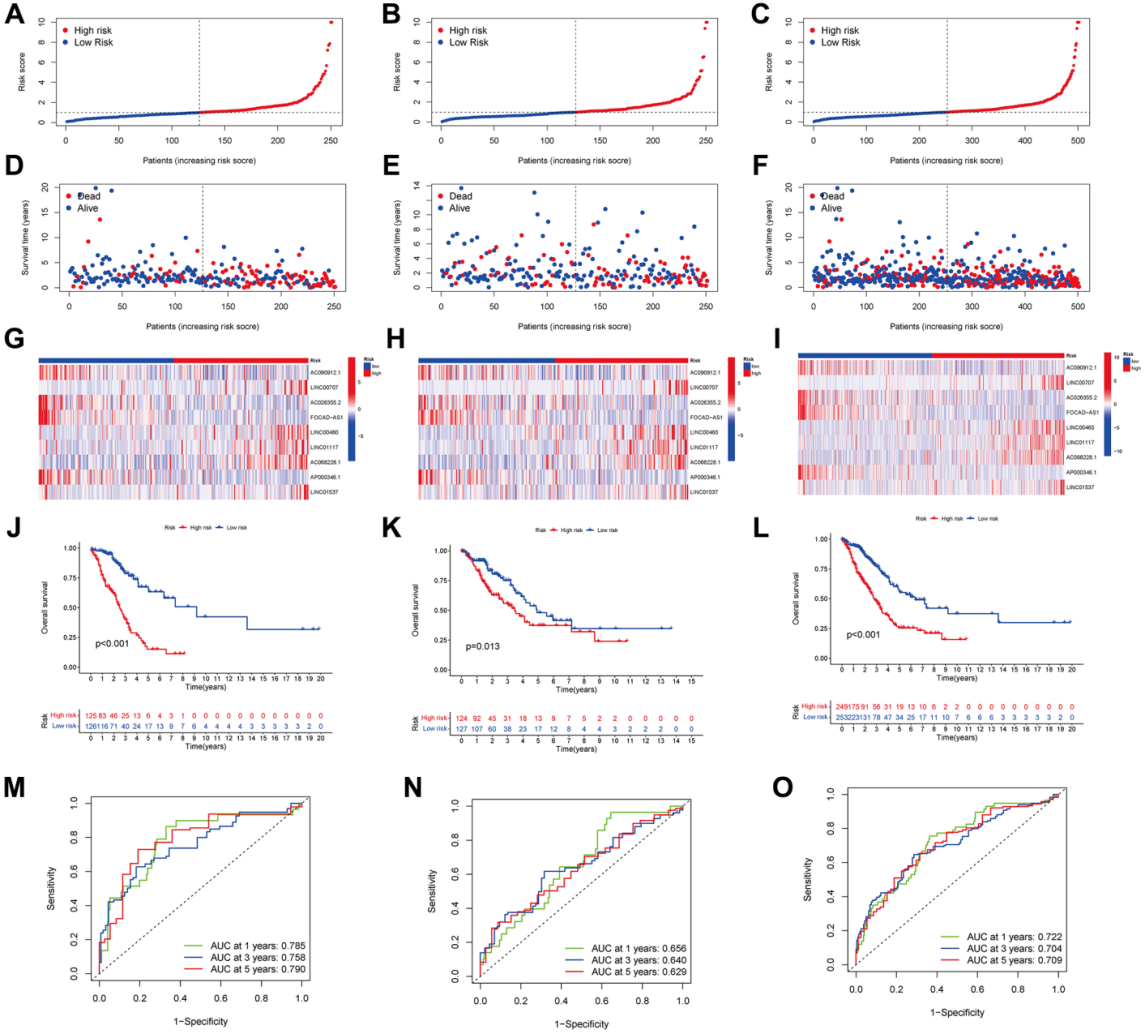
**Predicting the performance of characteristics.** The distribution of LUAD patients with varying risk scores in the training, testing, and entire sets is shown by the risk curves (**A**–**C**). The training, test, and entire set survival statuses of patients with differing risk scores (**D**–**F**). lncRNA characteristics in various datasets are shown by the heatmap (**G**–**I**). The three groups—training, testing, and the entire groups—are demonstrated via K-M curves that illustrate the overall survival (OS) of LUAD patients (**J**–**L**). The ARLSig’s predictive power for 1, 3, and 5-year OS in LUAD patients was demonstrated by the time-dependent ROC curves (**M**–**O**).

Each patient was assigned a risk score depending on a previously indicated formula. Subsequently, they were categorized into high- and low-risk groups using the median score as the dividing line. (Figure 4A–4C) illustrates the distributions of patients categorized as low- or high-risk for LUAD in the training, testing, and entire cohorts, and (Figure 4D–4F) illustrates the survival states. The results indicated that the high-risk patients exhibited a higher mortality rate than low-risk patients. lncRNAs characteristics in various cohorts are depicted on the heatmap (Figure 4G–4I). We identified LINC01117, AC068228.1, LINC00460, LINC00707, and LINC01537 as lncRNAs associated with high risk, while the remaining four were identified as lncRNAs associated with low risk.

A negative relationship between the risk score and the overall survival (OS) rate was noted in all three groups, as illustrated in (Figure 4J–4L) (*p* < 0.001, *p* = 0.013, and *p* < 0.001, respectively). Additionally, the signature-based area under the ROC curves (AUC) (Figure 4M–4O) for 1, 3, and 5 years, was > 0.600, with respective values of 0.722, 0.704, and 0.709 for the entire cohort, signifying strong discrimination performance across the training, testing, and entire cohorts.

**Figure 5:**
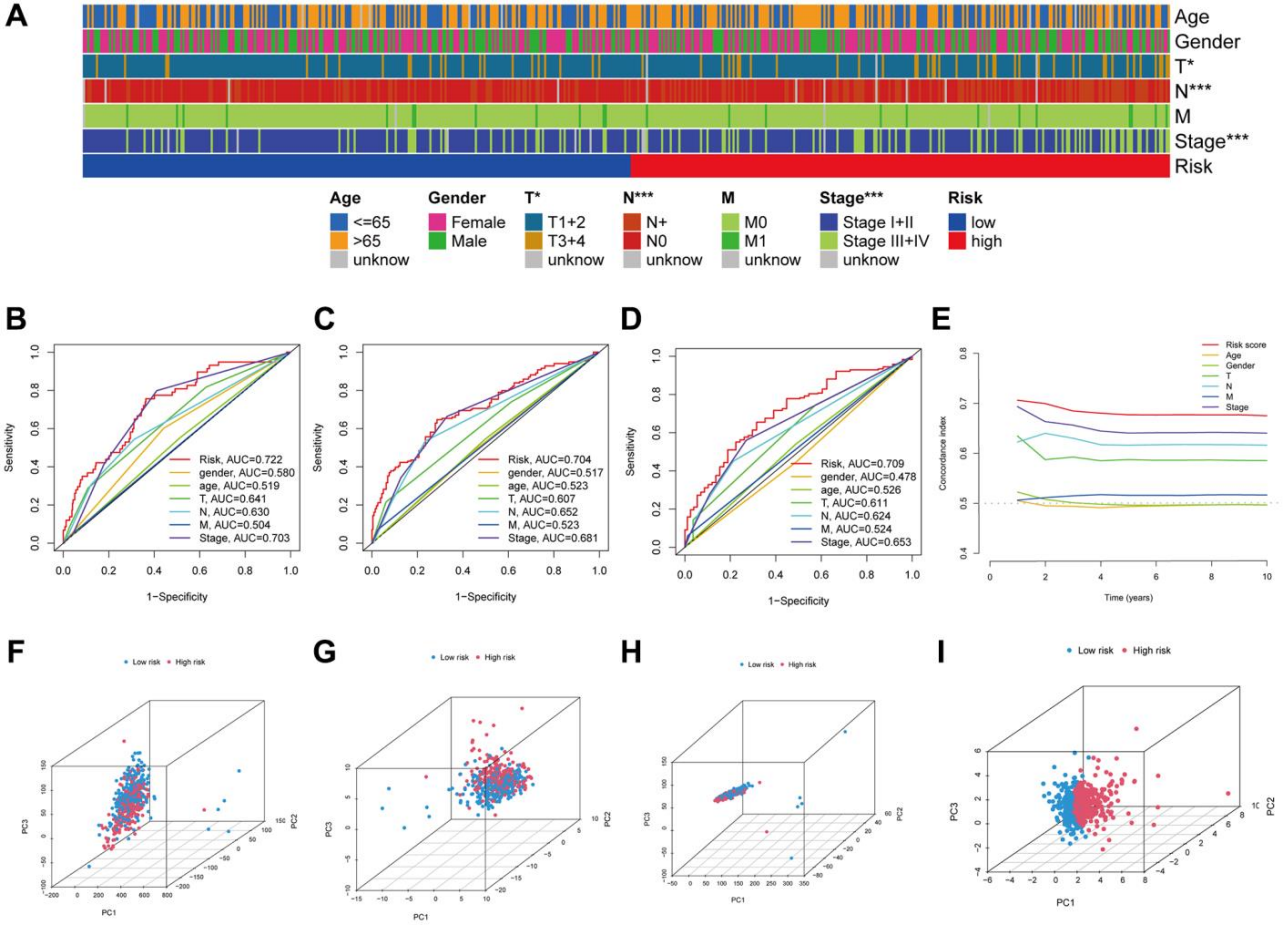
**The LUAD signature’s predictive power.** Visual representation of the variations in clinical characteristics between the high- and low-risk groups using a heatmap (**A**). The time-dependent ROC curves illustrate the risk score and clinical features’ predictive power for 1, 3, and 5 years OS (**B**–**D**). The risk score's concordance index was examined using C-index curves (**E**). Principal component analysis (PCA) in all genes, anoikis-related genes, anoikis-related lncRNAs and risk-related lncRNAs (**F**–**I**).

Next, we investigated how LUAD patients’ risk scores were correlated with their clinical characteristics. Figure 5A shows a heatmap visualization of the associations between high-risk scores and tumor stages (stages III–IV vs. I–II, *p* = 0.001), T stages (T3+4 vs. T1+2, *p* = 0.023), and N stages (N+ vs. N0, *p* = 0.001). In addition, the risk score, age, sex, and stage were employed to generate time-dependent ROC curves for OS prediction at 1, 3, and 5 years (Figure 5B–5D). Notably, the risk score’s concordance index (C-index) was close to 0.7 (Figure 5E), demonstrating strong predictive ability when contrasted with conventional clinicopathological markers. PCA was implemented for all genes, anoikis-related genes, anoikis-related lncRNAs and risk-related lncRNAs (Figure 5F–5I), and the findings proved that the signature was distributed clearly, which is a positive indicator that the ARLR signature is effective.

**Figure 6:**
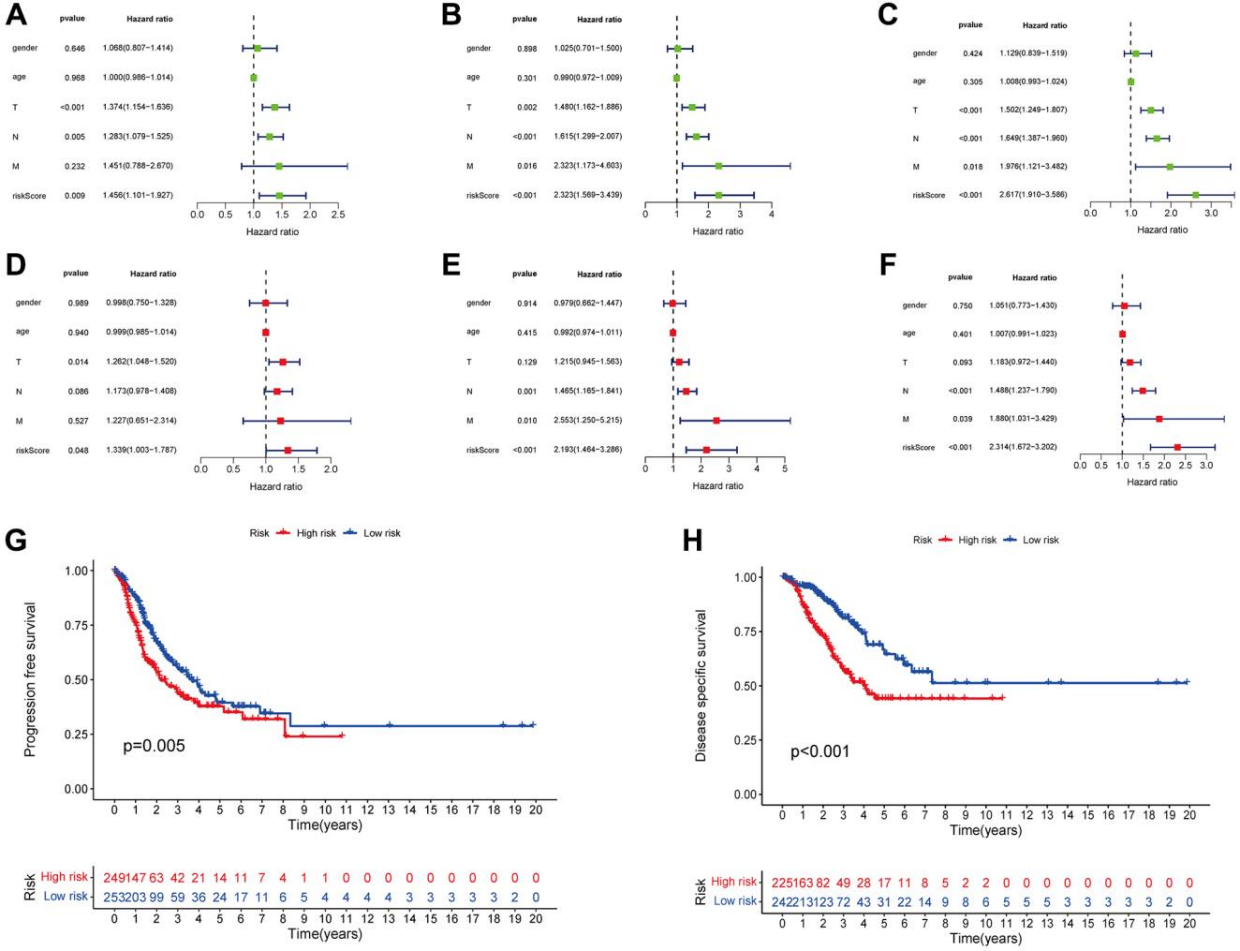
The risk score was correlated with PFS (**A**), DSS (**B**), and OS (**C**) as determined by univariate analysis, and multivariate analyses showed independently correlation between risk score and PFS (**D**), DSS (**E**), and OS (**F**). Variations in PFS and DSS between the groups with high- and low-risk scores are shown by the KM curves (**G**, **H**).

Comprehensive univariate and multivariate analyses of the entire cohort showed a robust link between the signature risk score and PFS, DSS, and OS (Figure 6A–6F). Notably, patients categorized as high-risk had a lower PFS compared to those categorized as low-risk (*p* = 0.005, Figure 6G). There was a substantial reduction in the DSS rate among individuals at high risk (*p* < 0.001, Figure 6H). This provided more evidence that the risk signature is an accurate indicator of LUAD patients’ prognoses.

### Nomogram development and assessment using the risk score

**Figure 7:**
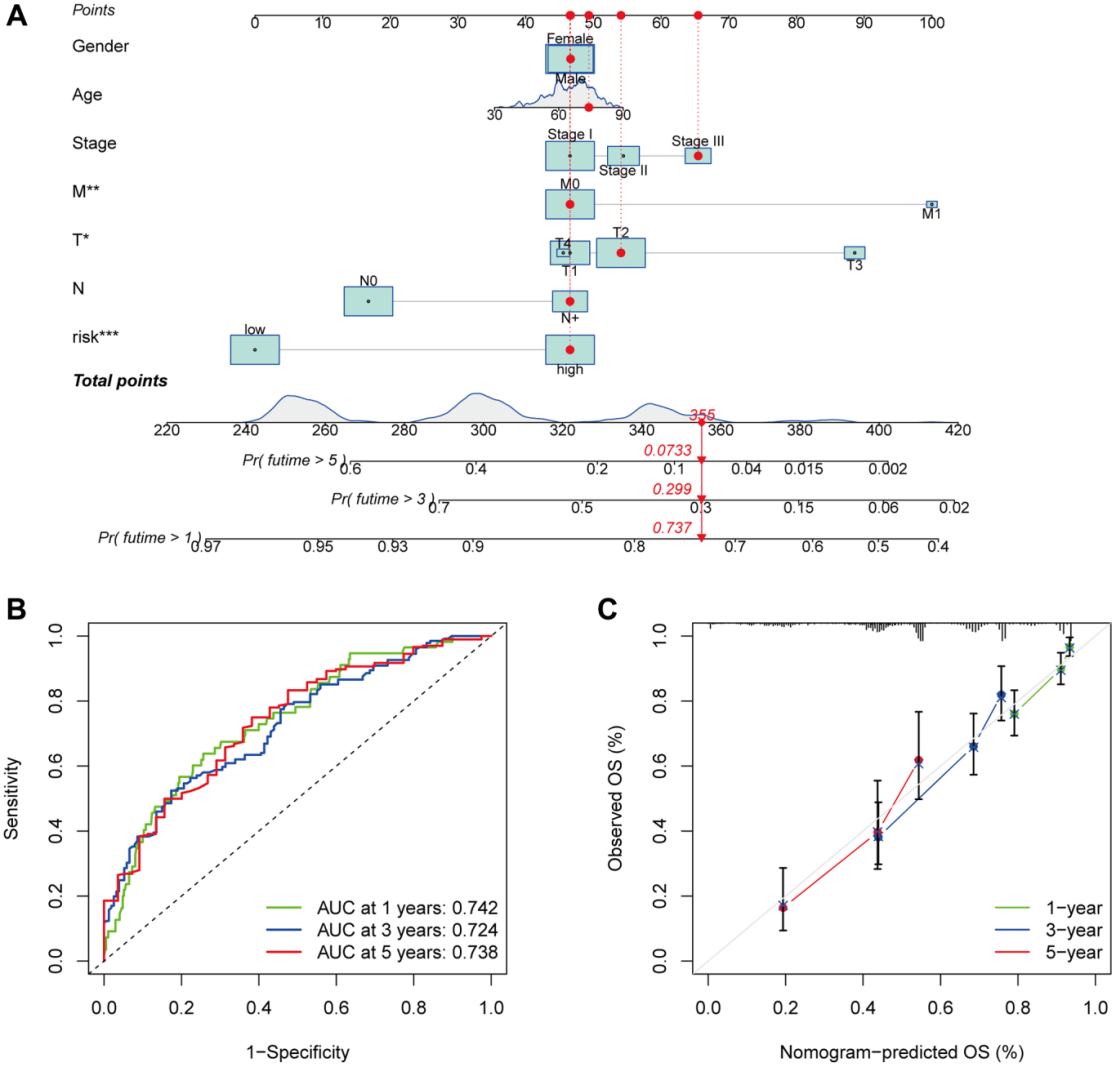
**Survival and prognostic prediction using nomogram and clinical features in LUAD patients.** An OS prediction nomogram for LUAD patients (**A**). The nomogram’s prognostic value was shown by the time-dependent ROC curves (**B**) and calibration curves (**C**) for 1, 3, and 5-year OS.

The nomogram was developed by evaluating the risk score in conjunction with other clinical parameters, which include age, sex, and T, N, and M stages (Figure 7A). The model’s reliability was evaluated by creating time-dependent ROC curves at 1, 3, and 5 years of follow-up, with corresponding AUCs of 0.742, 0.725, and 0.738 (Figure 7B). Furthermore, the nomogram forecasts and actual values were found to be in excellent agreement according to the calibration curves (Figure 7C). This suggests that the nomogram model is more useful in clinical settings for patient prognostic prediction than the risk score alone.

### Functional enrichment analysis

**Figure 8:**
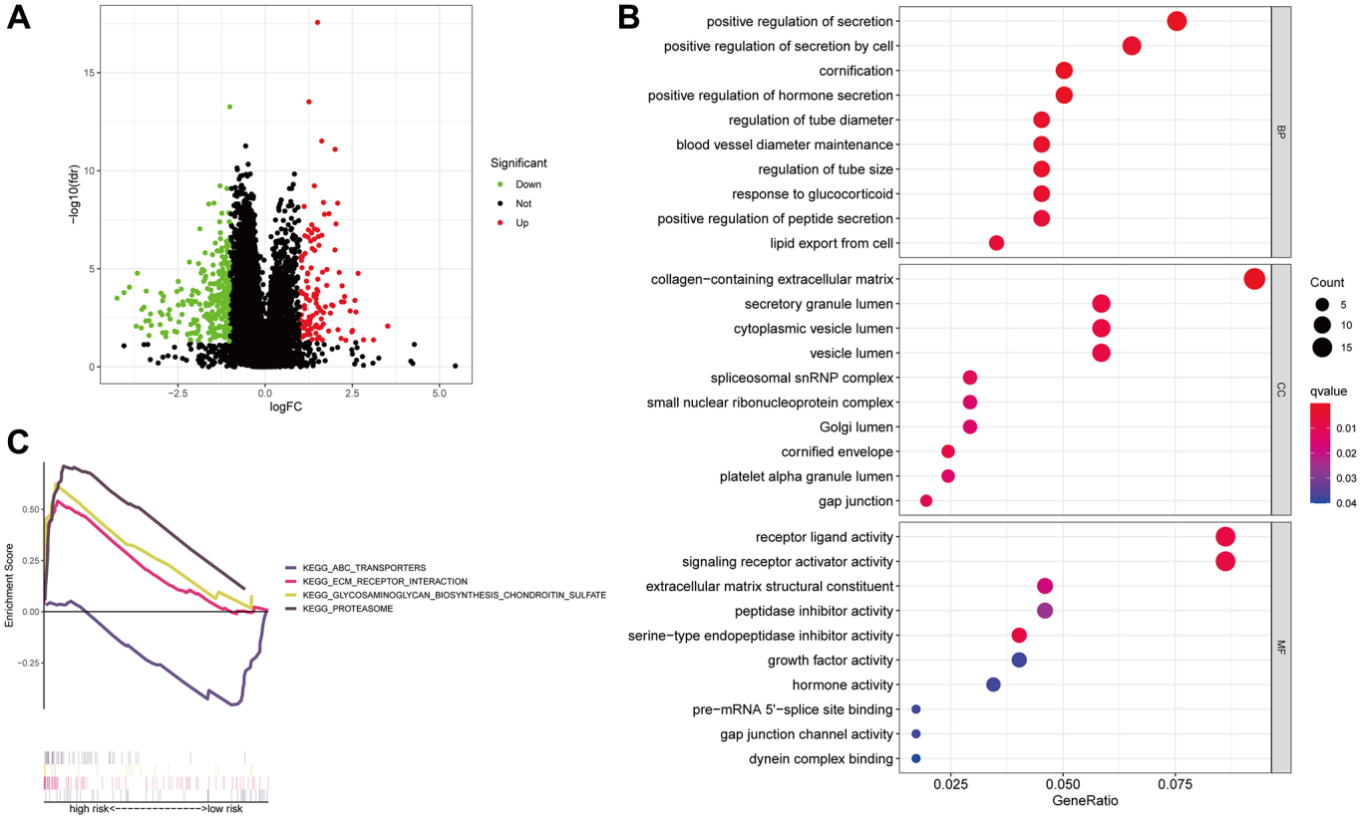
**Anoikis-associated lncRNA signature functional enrichment.** A volcano diagram illustrating the differential expression of genes (DEGs) among patients with LUAD (**A**). The GO analysis-derived bubble chart of DEGs between the two groups (**B**). GSEA-illustrated functional annotation between high- and low-risk groups (**C**).

We performed coexpression analysis between protein-coding genes and lncRNAs from the LUAD cohort and visualized the results in a volcano plot to learn more about the biology of the signature’s anoikis-related lncRNAs (Figure 8A). The GO study also showed that genes associated with anoikis could be involved in several of biological processes (BPs), cellular components (CCs) and molecular functions (MFs), which include positive regulation of secretion, receptor-ligand activity, collagen-containing extracellular matrix (ECM) organization, and signaling receptor activator activity (Figure 8B). To examine the pathways enriched in the Kyoto Encyclopedia of Genes and Genomes (KEGG) across the various categories, GSEA was implemented. The results illustrated that the PROTEASOME GLYCOSAMINGLYCAN BIOSYNTHESIS CHONDROTIN SULFATE, and ECM RECEPTOR INTERACTION, were predominantly enriched in the high-risk group (FDR < 0.05), whereas the ABC TRANSPORTERS pathway showed significant enrichment in the low-risk patients (FDR < 0.05) (Figure 8C).

### TMB correlation with the risk score

**Figure 9:**
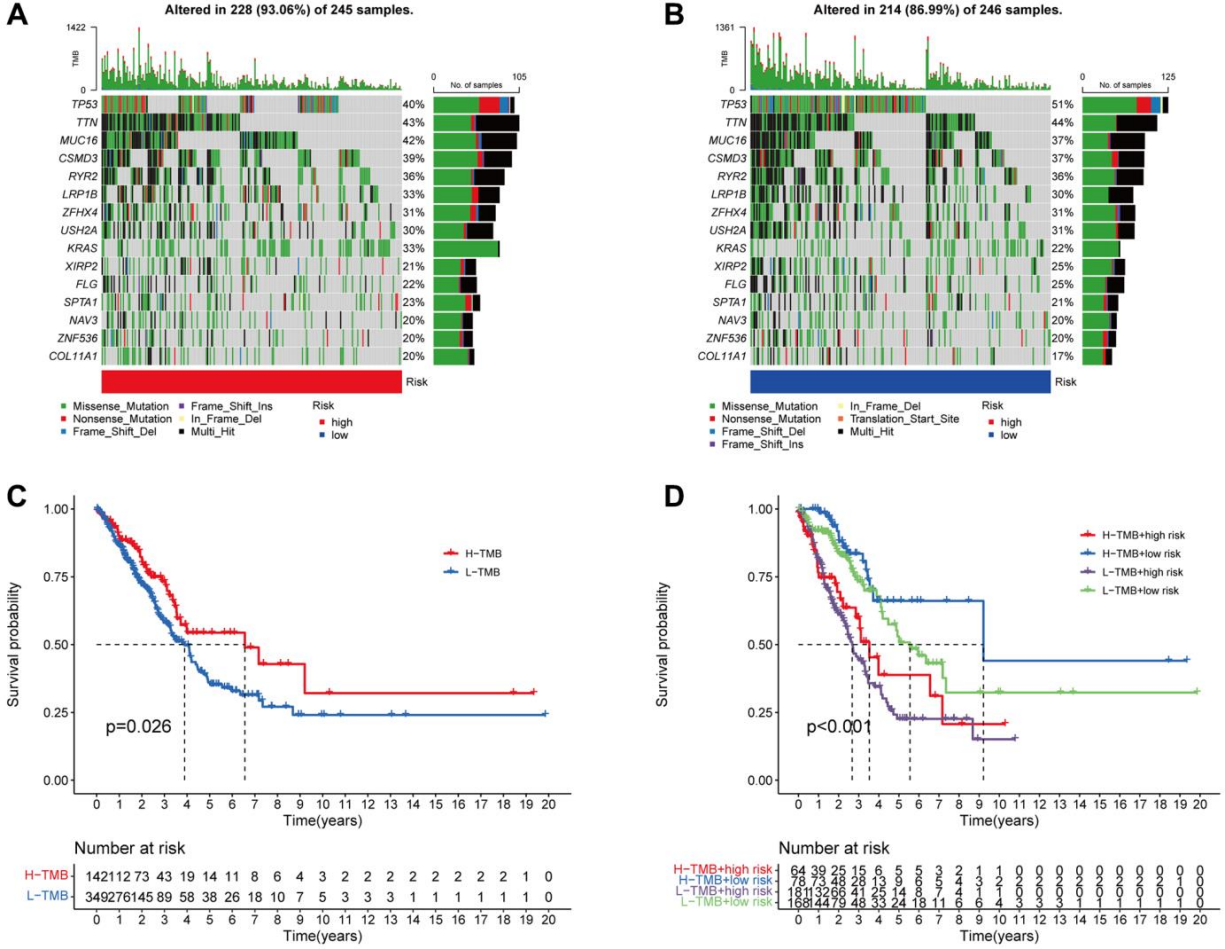
**The association between the signature and the TMB.** The 15 genes that are mutated most frequently in high-risk (245) and low-risk (246), LUAD patients, are displayed in the waterfall plot (**A**, **B**). Survival curves (**C**) and (**D**) illustrate the survival probabilities in groups with high and low TMB risks, and a combined TMB-risk survival curve, respectively.

In addition, 15 tumor mutations were identified in the high- and low-risk groups, and we demonstrated that the frequency of TP53 mutations was higher in the low-risk patients (high-risk: 40%, low-risk: 51%), whereas the high-risk patients had a greater frequency of KAS mutations (high-risk: 33%, low-risk: 22%, Figure 9A, 9B). According to survival analysis, patients who have a high TMB may experience a reduced duration of survival in comparison to those with a low TMB (*p* = 0.026, Figure 9C). We subsequently examined survival in terms of TMB and risk scores, and the groups with high TMB and low risk exhibited the best OS (Figure 9D).

### Immune function and screening for anticancer drugs

**Figure 10:**
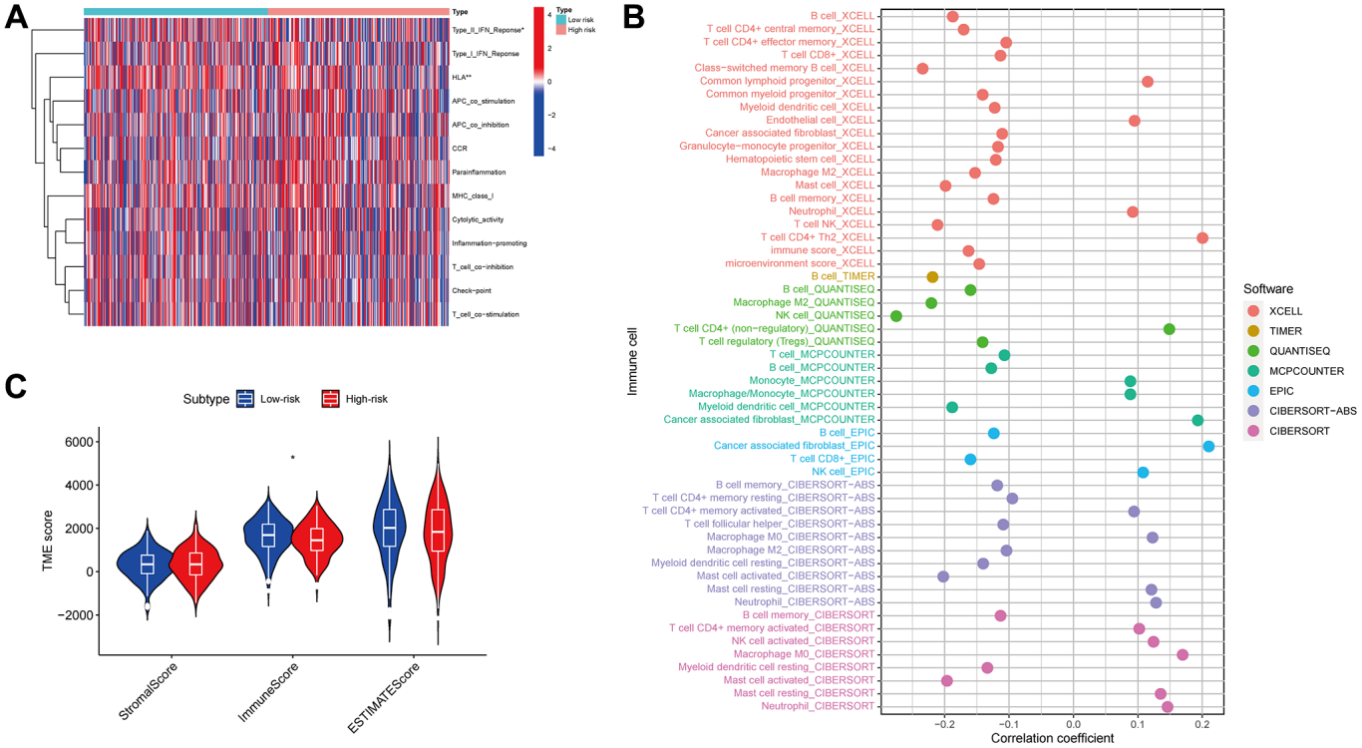
**Immune function.** Immunological functions of the high- and low-risk groups are depicted on the heatmap (**A**). The association of immune cells with the risk score (**B**). The correlation between the risk and TME scores (**C**).

An analysis of immune-related functions revealed, via heatmap, that the low-risk patients had significantly more active type II interferon (IFN) and HLA as opposed to those at high risk (Figure 10A). Furthermore, an extensive array of immune cells exhibited a negative association with the risk score, as determined by the evaluation conducted by various platforms (for example, B cells and CD8+ T cells in EPIC) (Figure 10B). Furthermore, it was observed that the immune score of the low-risk patients exceeded that of the high-risk patients. However, the stromal and ESTIMATE scores showed no significant variation between the two groups (Figure 10C). The findings suggest that immune-associated functions may be more prevalent in low-risk patients than those in high-risk individuals.

**Figure 11:**
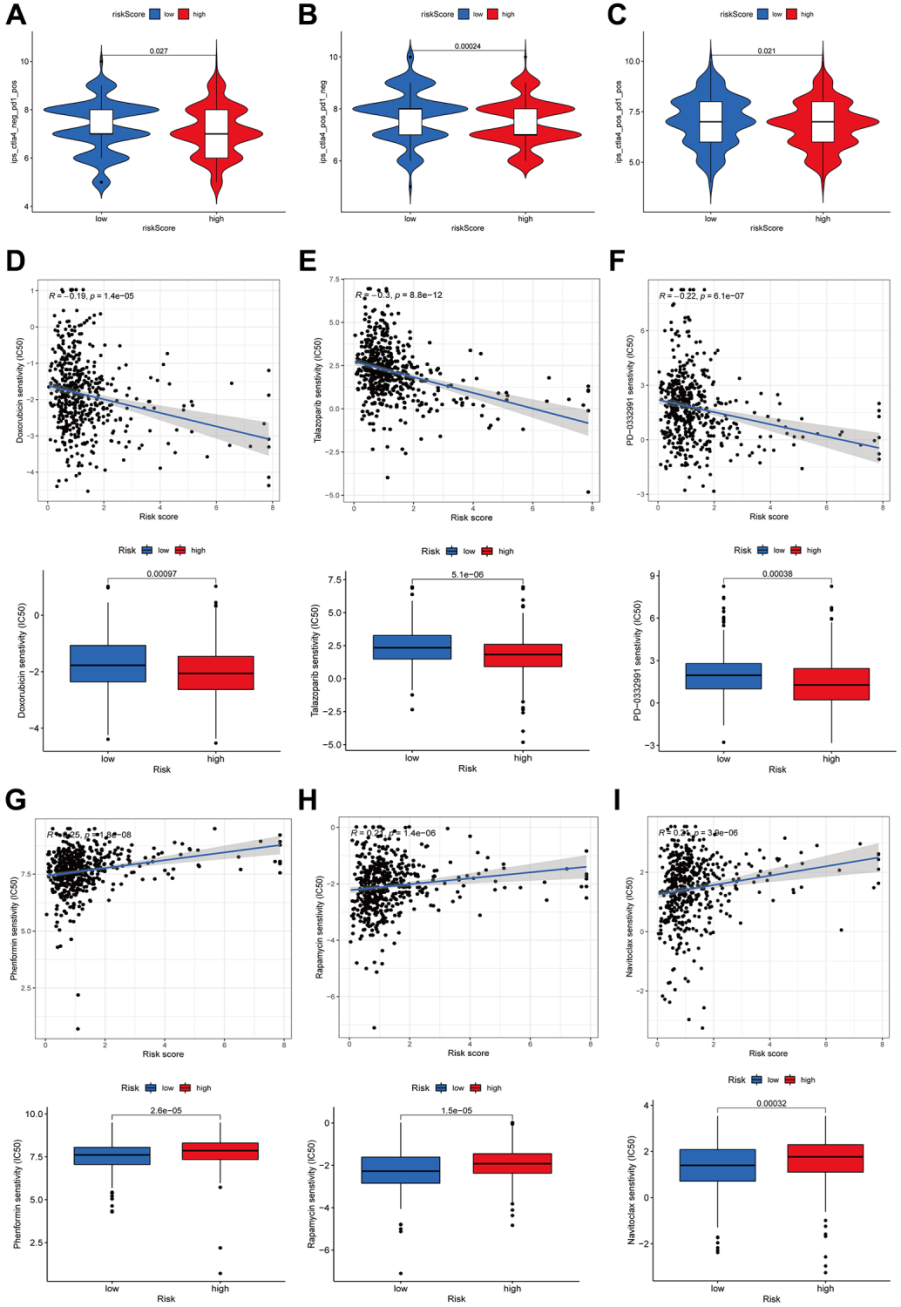
**Immunotherapy and drug sensitivity.** Correlations between the risk score and immunophenoscore (IPS) for patients receiving anti-PD1 alone (**A**), patients receiving anti-CTLA4 alone (**B**), and patients receiving combined anti-PD1 and anti-CTLA4 treatments (**C**). The drug sensitivity to doxorubicin (**D**), talazoparib (**E**), palbociclib (**F**), phenformin (**G**), rapamycin (**H**), and Naviroclax (**I**).

An investigation was conducted in LUAD patients to assess the link between risk scores and immunotherapy efficacy to examine the impact of the risk score on antitumor immune response. As demonstrated by violin plots derived from the IPS, low-risk score patients exhibited improved responsiveness to PD1 inhibitor therapy alone (*p* = 0.027, Figure 11A), CTLA4 inhibitor monotherapy (*p* = 0.00024, Figure 11B), and combined PD1 and CTLA4 inhibitors (*p* = 0.021, Figure 11C). Then, we determined the IC50 of several anticancer medications utilizing the pRRophetic package. These drugs included doxorubicin, talazoparib, palbociclib, phenformin, rapamycin and naviroclax. The findings demonstrated that doxorubicin, talazoparib, and palbociclib exhibited greater IC50 values in the low-risk patients contrasted with the other three medicines (Figure 11D–11I). Based on these results, the risk signature may be useful for guiding clinical anticancer treatment.

### Further exploration of AC026355.2

**Figure 12:**
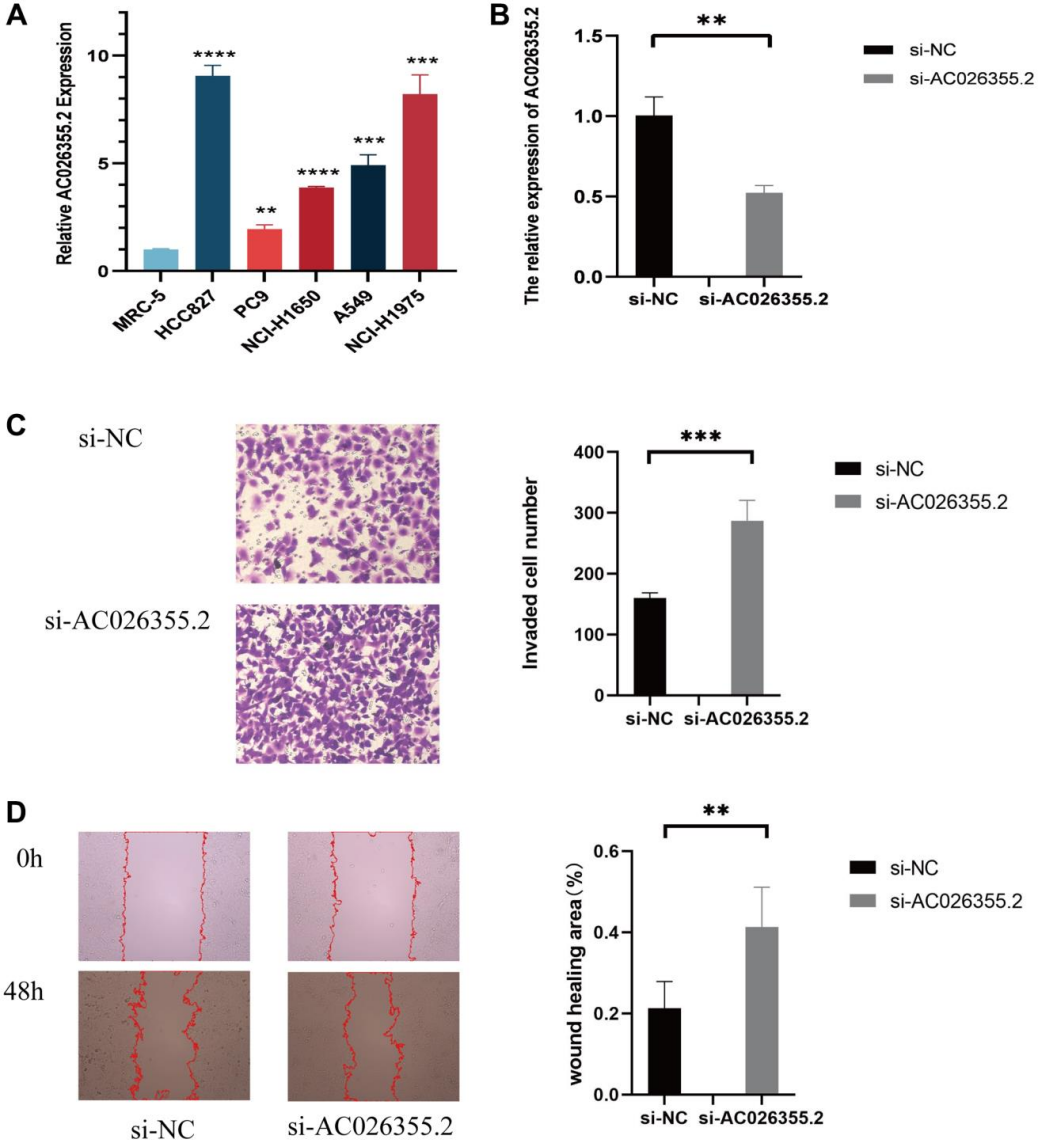
qPCR results showing the expression of AC026355.2 in adenocarcinoma cell lines (**A**). Relative AC026355.2 expression after siRNA transfection in A549 cells (**B**). Transwell (**C**) and scratch (**D**) assays for cell migration and invasion in A549 cells.

To ascertain the role of these lncRNAs in LUAD development, we selected AC026355.2, which has rarely been reported, for further study. We first evaluated the anoikis-related lncRNA AC026355.2 expression in cell lines. Consistent with our hypothesis, AC026355.2 was more highly expressed in adenocarcinoma cell lines (HCC827, PC9, NCI-H1650, A549, and NCI-H1975) (Figure 12A) than in the human embryonic lung cell line MRC-5. Afterward, we transfected AC026355.2 siRNA into the human lung cancer cell line A549. The qRT-PCR results demonstrated that the AC026355.2 expression was substantially decreased in the treated group than in the negative control group (Figure 12B). Then, we used transwell and scratch assays to assess the role of AC026355.2 in LUAD cell migration and invasion. According to the results of the transwell assay, AC026355.2 silencing markedly increased the number of A549 cells that infiltrated the bottom chamber (Figure 12C). Similarly, the scratches persisted in their healing process, and silencing AC026355.2 enhanced the cells’ capacity to migrate into the scratch region (Figure 12D).

## DISCUSSION

At present, lung cancer ranks first among cancers in terms of morbidity and mortality worldwide, Among the most prevalent types, LUAD is characterized by an abnormally high risk of lymph node metastasis, frequent distant metastases, and a negative prognosis [14]. For this reason, developing an accurate lung cancer risk profile to predict LUAD patients’ outcomes is paramount. lncRNAs are a subset of RNAs that are >200 nucleotides yet do not code for proteins [15]. LncRNAs, which are aberrantly expressed in NSCLC tissues, have a vital function in regulating the capacities of tumors to proliferate, invade, migrate, and undergo apoptosis. An upregulation of lncRNA XLOC_008466 was demonstrated in NSCLC patients [16]. Suppressing XLOC_008466 expression promotes cell apoptosis by inhibiting invasion and proliferation. The miR-874 expression may be downregulated by XLOC_008466 when it binds directly to it, but the XIAP and MMP2 expression, which are downstream targets, is upregulated, i.e., the carcinogenic effects of XLOC_008466 are exerted via the miR-874-MMP2/XIAP pathway, which impacts cell proliferation and invasion. Related research has additionally examined lncRNAs as potential key elements in lung cancer prognostic assessments. Tang et al. [17] investigated how the lncRNA expression is correlated with LUAD prognosis and discovered that five lncRNAs (ZNF503-AS1, RP11-54H7.4, RP11-38M8.1, RP11-108M12.3, and CYP4F26P) were linked to LUAD prognosis, with upregulation of RP11-54H7.4, RP11-38M8.1, RP11-108M12.3, and CYP4F26P, in tissue samples from lung cancer and downregulation of ZNF503-AS1, and the AUC to predict the 5-year OS of patients with these 5 lncRNAs was 0.691. The detachment of ECM-bound cells initiates anoikis, a distinct type of programmed cell apoptosis. The loss of cell adhesion to the ECM results in the detachment of the cytoskeleton-bound proapoptotic protein Bmf from kinesin light chain 2 and its subsequent translocation to the mitochondria, thereby facilitating anoikis [18]. Cancer cells are insensitive to anoikis and do not need to adhere to the ECM to survive and proliferate, and this ability has important implications for the metastatic process [7]. Notably, resistance to anoikis is an essential requirement for the aggressive metastasis of malignancy. Eun Young Kim et al. [19] showed that CEACAM6 upregulation in LUAD led to the activation of the src-FAK signaling pathway and induction of induced anoikis. Nevertheless, it is necessary to carry out additional research on the coregulatory function of lncRNAs and anoikis in LUAD.

We discovered lncRNAs correlated with anoikis by analyzing the coexpression of these genes in our study. Overall, 9 prognostic lncRNAs linked to anoikis, namely, AC090912.1, LINC00707, AC026355.2, FOCAD-AS1, LINC00460, LINC01117, AC068228.1, AP000346.1, and LINC01537, LINC00707, and AC090912.1, were identified utilizing LASSO regression and univariate analyses to build the prognostic signature. Only two of the nine lncRNAs associated with anoikis in the LUAD signature—LINC00707 and LINC00460—have had their cancer-related functions and underlying mechanisms reported. Multiple tumor types, which include colorectal, cervical, lung, and breast malignancies, have an increased expression of LINC00707. LINC00707 is implicated in LUAD in the regulation of cellular proliferation, migration, and apoptotic activity, by binding to miR-338-3p or enhancing Cdc42 expression, which increases AHSA1 levels [20].

In breast cancer, LINC00707 promotes CTHRC1 expression by inhibiting miR-30c, thereby enhancing tumor proliferation, invasion, and migration [21]. Hua Guo et al. [22] illustrated that LINC00707 facilitates the advancement of cervical cancer through the regulation of the miR-382-5p/VEGFA pathway. Furthermore, Huifang Zhu et al. [23] highlighted that miR-206 is sponged by LINC00707, thereby facilitating the proliferative potential and metastatic progression of colorectal cancer cells. Mounting evidence suggests that LINC00460 functions as a prominent regulator in carcinogenesis and is abundantly expressed as an oncogene in numerous types of malignancies. Research has demonstrated that LINC00460 facilitates the progression of head and neck squamous cell carcinoma (HNSCC) through the sponging miRNAs (miR-206, miR-612, miR-320a, and miR-4443) and inhibiting their expression [24–27]. Additionally, Yue QY et al. [28] demonstrated a significant upregulation of LINC00460 in NSCLC tissues, of which this overexpression was strongly correlated with pathological lymph node metastasis, TNM stage, and unfavorable NSCLC patients’ prognoses. One way via which LINC00460 contributes to apoptosis is by regulating the levels of apoptotic proteins which include cleaved caspase-2, Bax, Bcl-2, and PI3K/AKT [29]. LINC00460 influences cellular proliferation by sponging miR-539 and enhances cell migration and invasiveness via EMT-related genes [30]. Ye et al. [31] discovered that to stimulate LUAD cell proliferation, LINC00460 bound to miR-302c-5p and increased FOXA1 expression. Additionally, LINC00460 was shown to be upregulated in gastric cancer and was correlated with dismal patient prognoses. According to this study’s findings, LINC00460 influences gastric cancer cell proliferation and apoptotic activity via the downregulation of CCNG2 and recruiting LSD1 and EZH2 [32]. Few studies have focused on the other seven lncRNAs, thus much remains unknown about how they contribute to tumorigenesis and their specific mechanism.

A robust correlation was noted between the OS of LUAD patients and this 9-ARLSig in our study. The AUC was 0.79, 0.629, and 0.709 for predicting 5-year OS in the training, test, and entire groups, respectively. Based on a comparison with other clinical markers, this risk score exhibits a more significant capacity for prediction. Furthermore, univariate and multivariate analyses were executed on the risk scores, indicating that they function independently as prognostic factors for both PFS and DSS. As of now, no comprehensive reports of DSS have been identified in alternative lncRNA-related models utilized for prognosticating LUAD. Furthermore, we employed ROC curves and calibration curves to validate the nomogram based on the risk score, which exhibits a robust prognostic value for LUAD individuals. In summary, this risk signature exhibits a favorable prognostic capacity in patients with LUAD.

Analysis of functional enrichment was undertaken to investigate the possible mechanisms and functions of the lncRNAs in ARLRs. Genes associated with anoikis were substantially enriched in several functions, including the organization of the ECM-containing collagen, according to a GO analysis. Lung cancer-related genetics and epigenetics could play a role in the misexpression of integrins, proteases, and collagen in the tumor microenvironment (TME), thereby influencing the transformation of the ECM and potentially facilitating the advancement of the tumor [33]. Moreover, anoikis-resistant tumor cells are capable of proliferating and surviving without attaching to the ECM. Furthermore, differential KEGG pathway enrichment was identified between the high- and low-risk groups via GSEA. An increased enrichment of tumor-related pathways was recorded for the high-risk group, such as ECM RECEPTOR INTERATION, GLYCOSAMINGLYCAN BIOSYNTHESIS CHONDROTIN SULFATE and PROTEASOME, whilst the ABC TRANSPORTERS pathway enrichment was observed in the low-risk group.

Next, we examined 15 common tumor mutations in both high- and low-risk patients. Notably, the findings illustrated that the high-risk patients exhibited a reduced mutation rate of TP53 in comparison to those at low risk. Conversely, the high-risk patients demonstrated a higher mutation rate of KRAS. The present findings diverge from those of previous studies [34, 35]. A higher TMB was indicative of a prolonged OS, although TMB was shown to not correlate with risk score (*p* = 0.026). In many types of malignancies, particularly melanoma, lung cancer, and bladder cancer, TMB is commonly employed as a predictive biological marker for immune checkpoint blockade [36–38]. We hypothesize that tumor changes in many pathways impacting carcinogenesis and metastasis may be associated with the development of anoikis resistance, and additional research is required.

Additionally, we measured the immune-associated gene functions in both the high- and low-risk populations and discovered that high-risk patients had a suppressed type II IFN response. Among their many functions, the pleiotropic cytokines known as interferons (IFNs) exhibit immunoregulatory, antitumor, and antiviral properties. The immune response is mostly coordinated by IFNs, and IFNγ is the only gene product that makes up the type II IFN family. Multiple cell types, including T, NK, Treg, and B cells, can secrete IFNγ in the TME. Immunological cells in the TME may be influenced by IFNγ, leading to anticancer effects which include killing tumor cells, effector functions, cell migratory rate, proliferation of immune cells, and presentation of antigens [39]. Consequently, immune escape in high-risk patients may be due, in part, to the suppression of the type II IFN response, as shown in the current research. The TME is an essential component in immunotherapy, according to several previous studies [40]. The current research confirmed that the low-risk patients had a higher number of infiltrating immunological cells, including CD8+ T cells. A higher immune infiltration status is associated with improved immunotherapy efficacy [41]. Notably, CD8+ T cells could enhance the effectiveness of immunotherapy by attacking cancerous cells via the PD-1/PD-L1 immunosuppression axis and by breaking immunological tolerance [42]. After quantifying the TME based on the immune and stromal scores, we discovered higher immune scores in the low-risk group, thereby providing additional evidence that immunotherapy could be more effective in the low-risk population. After evaluating the risk score correlation with IPS, we determined that low-risk score patients exhibited a better chance of gaining benefit from immune treatment than those with high-risk scores, and this was true irrespective of whether PD1 or CTLA4 inhibitors were administered alone or in combination. Hence, we suggest that the model might be valuable for creating individualized and accurate treatment plans.

Furthermore, we compared the efficacy of some medications in high-risk and low-risk patients; we discovered that doxorubicin, talazoparib, and naviroclax were more effective in the former, but more inquiry into the exact mechanisms in which these treatments work and their impact on tumors is required. ARLsig is an invaluable resource for medication selection. Furthermore, we preliminarily explored the carcinostatic effect of AC026355.2 on LUAD cells and found that inhibiting AC026355.2 can increase the migration and invasion of tumor cells.

Despite the excellent prognostic value of our anoikis-related lncRNA signature, there are still several limitations to our research. Firstly, we only used data from one database, TCGA, for our analysis, and even though we separated the data into training and testing cohorts, the population still has to be validated. Secondly, we only chose a small number of clinical samples; hence, a bigger data set is required to evaluate the prediction model’s accuracy. Lastly, despite our investigation into the function of AC026355.2 in cell assays, the processes behind the majority of lncRNAs in the onset of cancer remain undetermined, and additional investigation using *in vitro* and *in vivo* tests is required to clarify the biological mechanism or prognostic significance of anoikis-related lncRNAs in LUAD.
